# Mechanism of dielectric barrier discharge plasma technology to improve the quantity of short-chain fatty acids in anaerobic fermentation of waste active sludge

**DOI:** 10.3389/fmicb.2022.963260

**Published:** 2022-07-22

**Authors:** Jie Wang, Xingguo Liu, Jinling He, Guofeng Cheng, Junli Xu, Ming Lu, Yuyi Shangguan, Ai Zhang

**Affiliations:** ^1^Fishery Machinery and Instrument Research Institute of Chinese Academy of Fishery Sciences, Shanghai, China; ^2^Key Laboratory of Aquaculture Facilities Engineering, Ministry of Agriculture and Rural Affairs, Shanghai, China; ^3^College of Environmental Science and Engineering, Donghua University, Shanghai, China; ^4^School of Ecology and Environment, Yellow River Conservancy Technical Institute, Kaifeng, China; ^5^School of Environment and Architecture, University of Shanghai for Science and Technology, Shanghai, China; ^6^School of Ecological and Environmental Sciences, East China Normal University, Shanghai, China

**Keywords:** anaerobic digestion, microbial community structure, sludge recycling, sludge carbon resource, plasma technology

## Abstract

The mechanism of improving the anaerobic fermentation performance of waste active sludge by using dielectric barrier discharge (DBD) plasma pretreatment technology was investigated. The maximum accumulation of short-chain fatty acids (SCFAs) was observed on the 7th day of anaerobic fermentation when the DBD power was 76.50 W, which was 1726.70 mg COD/L, 1.50 times of the control group. The ratio of acetic acid in DBD group was 9.30% higher than that in the control. Further mechanism research indicated that DBD pretreatment can destroy the structure of extracellular polymer substances and release organic substances such as protein and polysaccharide. The dissolved organic matter analysis indicated that the DBD technique could increase the release of biodegradable organics (eg., tyrosine proteins, soluble microbial by-products), thus accelerate the biotransformation of organic substance. Bacterial community structure analysis showed that the increase in the abundance of Firmicutes and Bacteroidetes and the decrease in the abundance of Proteobacteria in DBD group were beneficial to the accumulation of SCFAs. Besides, further archaeal analysis indicated that the decrease of *Methanosaeta* sp. and *Methanosarcina sp*. abundance in the DBD group facilitate acetic acid accumulation. This study demonstrated that the DBD technique can be used as an effective and potential pretreatment method to improve sludge anaerobic fermentation performance.

## Introduction

Waste activated sludge is a by-product of municipal sewage treatment plants, and its output is huge and increasing year by year (Jin et al., 2014; Liang et al., 2021). Sludge has the characteristics of high-water content, easy to decay, odor, and complex composition (including heavy metals, pathogenic microorganisms, refractory organic compounds, and other pollutants; Liang et al., 2021; Chen et al., 2022). If it is not treated timely and effectively, it will cause serious harm to the environment (Jin et al., 2014; Chen et al., 2018; Patel et al., 2021). Therefore, the safe, effective, and economic disposal of sludge has always been one of the major problems that need to be solved urgently in the environmental field (Jin et al., 2021; He et al., 2022). The technology of sludge resource utilization has a good application prospect (Lee et al., 2014; Wu et al., 2021). Anaerobic fermentation technology is a commonly used means of sludge recycling in recent years, that is, the process of microbial degradation of organic compounds and their conversion into recyclable carbon resources in an anaerobic environment (Luo et al., 2021, 2022; Yang et al., 2022). Anaerobic fermentation process has the advantages of low energy consumption, high organic load, and no pollution (Wang et al., 2021, 2022a; He et al., 2022). It can not only reduce the sludge, but also produce products with high added value, which can effectively promote the resource utilization of sludge (Liang et al., 2021; Wang et al., 2022b,c).

Short-chain fatty acids (SCFAs) are the intermediate products of anaerobic fermentation of sludge and are widely used in bioenergy, biomaterials, textiles, and other industries (Lee et al., 2014). SCFAs can be used as the substrate for microbial fuel cell power generation (Tharak and Mohan, 2020); in addition, SCFAs can be used to produce polymers (Jayakrishnan et al., 2020; Kumar et al., 2021) and hydrogen (Yesil et al., 2021). At present, SCFAs are mainly produced from petrochemical resources (Huang et al., 2002). However, the non-renewable nature of petrochemical resources and the continuous rise in prices have increased the production cost of SCFAs and brought a series of environmental problems (Akaraonye et al., 2010). Anaerobic fermentation with organic wastes can not only reduce the production cost of SCFAs, but also reduce the impact of organic wastes on the environment (Jin et al., 2014; Jiang et al., 2021b). So, the preparation of SCFAs by anaerobic conversion technology is a potential resource technology of organic waste.

However, the anaerobic fermentation technology of sludge has the disadvantages of long fermentation cycle and low organic matter conversion efficiency (Gonzalez et al., 2018; Xu et al., 2021). The main reason is that most of the organic substance is wrapped in the sludge floc and the cell wall (membrane) structure of microorganism, and the cell structure of microorganism is very stable (Pang et al., 2020b; Zhang et al., 2022). It is necessary to destroy the cell wall (membrane) to release the intracellular organic matter, so that the anaerobic microorganism can transform the organic matter (Jiang et al., 2021b). For the purpose of improving the efficiency of sludge anaerobic fermentation, various physical (e.g., microwave; Wang and Li, 2016), chemical (eg. persulfates; Fang et al., 2021), and biological (e.g., protease; Pang et al., 2020a) pretreatment technologies can be used to strengthen the destruction of sludge floc and cell structure and release the organic components in sludge, so as to improve the anaerobic fermentation performance and shorten the anaerobic fermentation cycle (Gonzalez et al., 2018; Jiang et al., 2021a).

In recent years, plasma technology, as a novel technology, has been applied to the field of sludge anaerobic fermentation pretreatment (Choi et al., 2006; Svarnas et al., 2020; Li et al., 2021; An et al., 2022). Plasma technology is a means of using partially ionized plasma produced by a plasma generator to achieve certain goals (Kanazawa et al., 2011; Sun et al., 2012). Plasma can be produced artificially by many methods (Svarnas et al., 2020; Li et al., 2021). The principle of plasma technology for pretreatment of sludge is that the collision of particles will produce a large number of active components such as various free radicals (HO•, HO_2_•, O_2_^−^•, NO_2_•), electrons (e^−^), and molecules (O_3_, H_2_O_2_, etc.; Choi et al., 2006; Svarnas et al., 2020; An et al., 2022). These active components can destroy the sludge structure and release more organic substances, so as to improve the biotransformation efficiency of organic compound in anaerobic digestion process. Among many plasma technologies, dielectric barrier discharge (DBD) has the advantages of long electrode life, low energy consumption, high efficiency, simple device, and so on, and has the prospect of large-scale application (Kovacevic et al., 2017; Wang et al., 2018). The principle of DBD technology is that the discharge channel of gas between metal electrodes is blocked through the insulating medium. When the electric field reaches a certain strength, the gas between electrodes will be broken down to form a randomly distributed filiform discharge, which will produce a large number of charged particles. These charged particles collide with O_2_, N_2_, H_2_O, etc., between electrodes, producing free radicals and active particles, accompanied by UV radiation and shock wave (Wang et al., 2018, 2022b; Zhu et al., 2022). DBD technology has been widely used in the degradation of organic pollutants (e.g., dimethyl phthalate, glucocorticoids) in aqueous solution, and has been proved to have an excellent degradation effect on organic pollutants (Liu et al., 2019; Guo et al., 2022), but it has not been studied as a pretreatment technology of sludge anaerobic fermentation. Therefore, it is of certain significance to study whether DBD technology can improve the anaerobic fermentation efficiency of sludge, so as to provide a novel and potential method for the recovery of sludge.

This paper studied the effect of DBD pretreatment on the anaerobic fermentation performance of sludge and analyzed the mechanism. Soluble chemical oxygen demand (SCOD), VSS solubilization rate (S_VSS_), and ammonia nitrogen (NH+ 4-N) were analyzed to evaluate the effect of discharge power of DBD on sludge solubilization. Then, the maximum accumulation and composition of SCFAs were detected to evaluate acid production performance. In order to clarify the mechanism of DBD pretreatment technology to increase SCFAs production by anaerobic fermentation, the effects of DBD treatment on the cracking of sludge extracellular polymer substances (EPS), the degradability of soluble organic matter, and the community structure of bacterial and archaeal were studied. The conclusion of this work can provide an effective and promising pretreatment technology for sludge anaerobic fermentation.

## Materials and methods

### Source and basic characteristics of sludge

The waste activated sludge is from the secondary sedimentation tank of a sewage treatment plant in Shanghai. After sedimentation and drainage, the retrieved sludge is filtered with a 20 mesh-sieve to remove large inorganic particles such as sand, and the separated supernatant is used to dilute the sludge to a certain concentration, so as to ensure that the initial concentration of each batch of experimental sludge is similar. The basic characteristics of each batch of sludge are listed in Supplementary Table S1.

### Chemical reagent

The water used in the test is ultrapure water, which is prepared by Mill-Q ultrapure water device (Mingche-D24UV, German). Other chemicals are analytical grade and come from Sinopharm Chemical Reagent (Shanghai, China).

### Dielectric barrier plasma equipment

**Figure 1:**
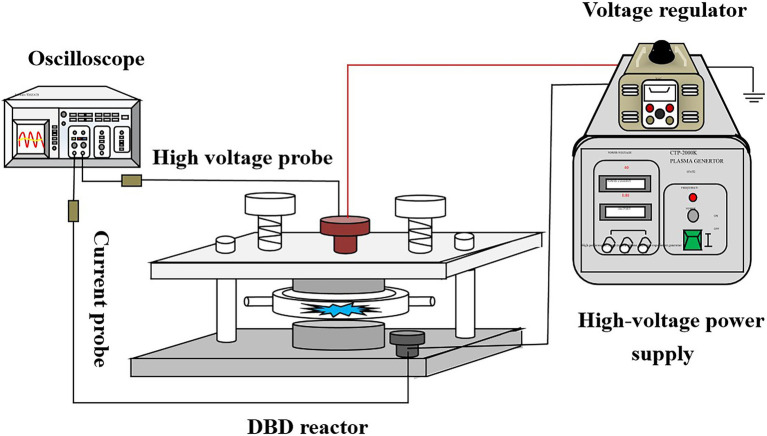
Schematic diagram of dielectric barrier discharge (DBD) equipment.

As shown in Figure 1, the DBD device is composed of a plasma high-voltage power supply, a dielectric barrier discharge reactor, an oscilloscope, and a voltage and current probe. Among them, the model of high-voltage plasma power supply is CTP-2000 K, which can convert the alternating current of a laboratory power distribution system (220 V/50 Hz) into high-voltage high-frequency alternating current (the maximum value can be 60 kV/20 kHz). The digital oscilloscope (TDS2012B) is connected to the discharged high voltage electrode and the ground electrode, respectively, for deriving voltage and current signals. The composition of the DBD reactor is described in Supplementary Material.

### Sludge pretreatment process

The reaction dish (inner diameter 150 mm and thickness 14 mm) is made of quartz medium, which has a large dielectric constant, accumulates more wall charges on the surface of the medium, and generates a large built-in electric field, which is conducive to the generation of uniform and stable discharge plasma. Place the sludge sample in the reaction dish (inner diameter of 150 mm, thickness 14 mm) and pretreatment under different DBD power (36.80, 76.50, and 113.20 W).

### Anaerobic fermentation experimental device

Take 400 ml of pretreated sludge and mix it with untreated sludge in the ratio of 7:1 as an anaerobic fermentation substrate. The anaerobic fermentation experimental device consists of a constant temperature incubator, a magnetic stirrer, and a series of 500 ml glass serum bottles with rubber soft plugs. Place the glass serum bottle with sludge sample and rotor on the magnetic stirrer, adjust the appropriate speed to make the vortex size in each bottle moderate and consistent, so as to mix the pretreated sludge and untreated sludge, and ensure that the fermentation substrate in the glass serum bottle is in a uniform state during the whole anaerobic fermentation process. Place the magnetic stirrer in a constant temperature incubator at 35°C to ensure the constant temperature of the whole anaerobic fermentation process. The sampling operation is completed in a constant temperature water bath with a magnetic stirring function to avoid temperature changes caused by sampling. Conduct nitrogen blowing on the glass serum bottle for 1 min after each sampling.

### Detection of short-chain fatty acids

A GC-2018 gas chromatograph (Shimadzu, Japan) is used for the analysis and determination of SCFAs. The model of chromatographic column is DB-FFAP (30 cm × 0.25 mm), and the carrier gas is nitrogen. After centrifugation, the supernatant was μM water MCE filter membrane filtration, add 3% formic acid to acidify the sample, and place it in a gas phase brown vial to test. The concentration of SCFAs is the sum of acetic acid, propionic acid, iso-butyric acid, butyric acid, iso-valeric acid, and valeric acid. The concentration of SCFAs is converted to COD, and the conversion coefficient is as follows: acetic acid: 1.067, propionic acid: 1.514, iso-butyric acid and butyric acid: 1.818, and iso-valeric acid and valeric acid: 2.039 (Li et al., 2008, 2014).

### Detection of extracellular polymer substances

Extraction of extracellular polymer substances (EPS) is divided into soluble extracellular polymer substances, loosely bound extracellular polymers (LB-EPS), and tightly bound extracellular polymers (TB-EPS). This study focuses on LB-EPS and TB-EPS and the extraction procedure is based on the method reported by Xu et al. (2013). The detailed extraction steps of EPS are shown in Text S2. The proteins in LB-EPS and TB-EPS were measured by the modified Lowry method using a bovine serum albumin standard (Li and Yang, 2007). Carbohydrates in LB-EPS and TB-EPS were analyzed by the anthrone-sulfuric acid method using a glucose standard (Felz et al., 2019).

### Determination of conventional indexes of sludge

The sludge sample was centrifuged and separated to obtain the supernatant, then the supernatant passed through a 0.45 μm filter membrane, then SCOD and NH+ 4-N were determined according to the standard method (APHA, 2006). Take V mL of sludge sample and centrifuge it for 20 min under the condition of 9,000 r/min, remove the supernatant, transfer the remaining residue to the crucible, put the crucible into an oven (105°C) and dry it to constant weight, record the mass as m_1_, then put the crucible into a muffle furnace (600°C) and burn it for 2 h, then weigh it, and record the mass as m_2_. VSS is calculated as follows: VSS = (m_1_-m_2_)/V, unit: mg/L. S_VSS_ can be calculated by the following formula:


$$S_{VSS}=\frac{VSS_{0}-VSS}{VSS_{0}}\times 100\%$$

where VSS_0_ represents the VSS of raw sludge.

### Detection of dissolved organic matter

Dissolved organic matter (DOM) in sludge fermentation broth was determined by a three-dimensional fluorescence spectrometer (F-4500FL spectrophotometer, Hitachi, Japan). Firstly, the sludge sample is centrifuged and separated to obtain the supernatant, and then the supernatant passes through 0.45 μm water MCE filter membrane filtration as the sample to be tested. Set the slit width of Ex and Em as 5 nm, the scanning range as 200–550 nm, the scanning speed as 2,400 nm/min, and the step size as 5 nm.

### Microbiological analysis

Microbial community structure was analyzed by high-throughput sequencing. Take 20 ml of sludge sample and put it into a sterilized spiral port centrifuge tube, centrifuge it for 15 min under the condition of 8,000 r/min, remove the supernatant, and conduct high-throughput sequencing on the solid-phase sludge after sealing. The primers for *bacteria* were 515F (5′-GTGCCAGCMGCCGCGG-3′) and 907R (5′-CCGTCAATTCMTTTRAGTTT-3′), and for *archaea* were 524F10extF (5′-TGYCAGCCGCCGCGGTAA-3′) and Arch958RmodR (5′-YCCGGCGTTGAVTCCAATT-3′). The DNA extraction, PCR amplification, and Illumina MiSeq sequencing data analysis were referred to in our previous study (Li et al., 2015). The raw sequencing data have been submitted to the NCBI Sequence Read Archive (SRA) under the accession number SRP370640.^1^

### Data analysis

All analyses are conducted three times and the data are presented as the mean ± standard deviation. The data of this experiment are mainly analyzed by Origin2021. Venn analysis was performed with R software to reflect the similarity between different samples. The Shannon index was calculated by MOTHUR.

## Results and discussion

### Effect of discharge power of dielectric barrier discharge on sludge solubilization

**Figure 2:**
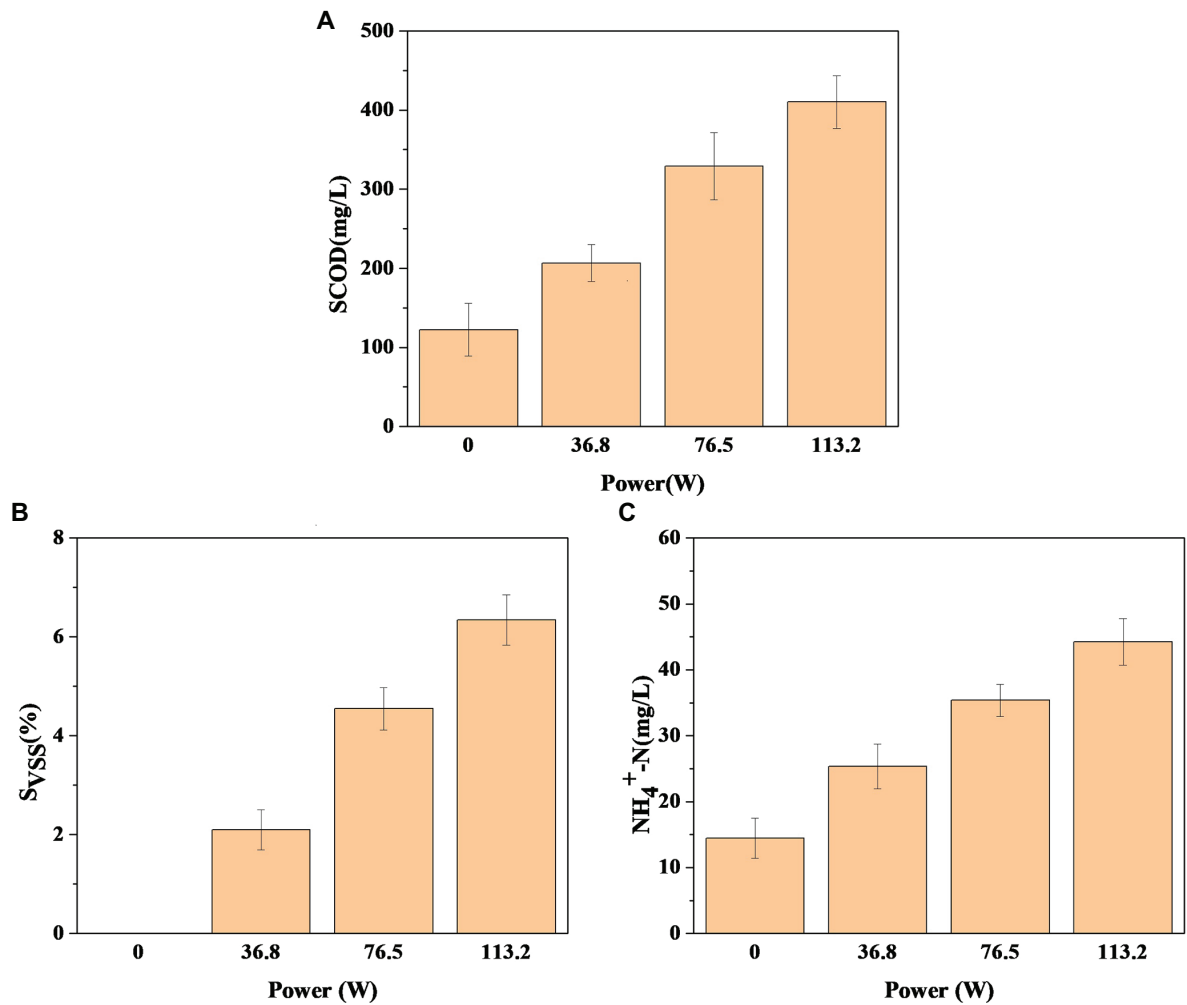
Changes in soluble chemical oxygen demand (SCOD) **(A)**, the VSS solubilization rate (S_VSS_) **(B)**, and ammonia nitrogen (NH+ 4-N) **(C)** after pretreatment (pretreatment time is 30 min).

To evaluate the effect of DBD discharge power on sludge solubilization, the pretreatment time was set as 30 min, and the changes of SCOD, S_VSS,_ and NH+ 4-N were investigated when DBD discharge power was 36.80, 76.50, and 113.20 W. Figure 2A shows the change of SCOD after DBD pretreatment of sludge. In Figure 2A, the dissolution amount of SCOD increases with the increase of discharge power. When the discharge power is 36.80, 76.50, and 113.20 W, the release amount of SCOD increases by 84.20 mg/l, 206.70 mg/l, and 287.80 mg/l, respectively, compared with the control group. SCOD is an important indicator to characterize the change of organic components in the anaerobic fermentation process of sludge. The dissolved organic matter in the pretreatment process can provide sufficient substrates for the hydrolysis and acidification stage of anaerobic fermentation. Therefore, the amount of SCOD dissolved after DBD pretreatment has a great impact on the fermentation process. The data results show that DBD pretreatment can strengthen the disintegration degree of sludge, release more organic matter, and lead to the increase of SCOD concentration in sludge, so as to provide more available substrates for the subsequent anaerobic fermentation process. In addition, when the discharge power is 36.80, 76.50, and 113.20 W, the S_VSS_ of sludge increases by 2.10, 4.60, and 6.30% respectively, indicating that the solubilization rate of volatile suspended solids gradually increases with the increase of discharge power (Figure 2B). This is consistent with the detection results of SCOD in Figure 2A, which indicates that DBD pretreatment promotes the transfer of organic matter in the solid phase of sludge to the liquid phase.

The main causes of DBD pretreatment leading to the solubilization of organic substances are as follows: (1) During the discharge process, the physical effects such as ultraviolet light, heat, and ultrasonic cavitation caused by electric shock destroy the extracellular polymer and microbial cell structure in sludge; (2) DBD will produce a large amount of •OH, H_2_O_2_, O_3_, etc. during discharge. These oxidizing substances can crack the structure of sludge polymer and release the organic substances in extracellular polymer and cells into the supernatant (Li et al., 2021; Zhu et al., 2022). With the increase of discharge power, the physical effect produced by a single discharge between the two plates increases, the concentration of high-energy electrons and active substances increases, and the cracking effect of sludge increases (Wang et al., 2018; Li et al., 2021).

In the sludge hydrolysis process, nitrogen-containing compounds are transferred from the solid phase to the liquid phase, resulting in a continuous increase of soluble nitrogen (Liang et al., 2021; Wu et al., 2021). Then, soluble nitrogen is hydrolyzed to peptides, dipeptides, amino acids, and even small molecular organic acids, ammonia nitrogen, and carbon dioxide, resulting in the increase of NH+ 4-N concentration in sludge supernatant (Liang et al., 2021; Wang et al., 2021). There are some anaerobic microorganisms in the sludge, so in Figure 2C, the concentration of NH+ 4-N in the supernatant of raw sludge was 14.50 mg/l, when the DBD discharge power was 36.80 W, 76.50 W, and 113.20 W, the concentration increased to 25.30, 35.40, and 44.20 mg/l, indicating that DBD pretreatment improved the hydrolysis effect of protein in sludge. In conclusion, DBD pretreatment is conducive to the solubilization and hydrolysis of sludge, and within the experimental power range set in this study, the solubilization and hydrolysis degree of sludge increase with the increase of discharge power.

### Effect of discharge power of dielectric barrier discharge on anaerobic fermentation process

DBD pretreatment can improve the crack of sludge, but the anaerobic fermentation property of pretreated sludge is not clear. Therefore, this study further compares the anaerobic fermentation property of sludge under different pretreatment conditions. The changes of SCOD, S_VSS,_ and NH+ 4-N of the sludge after 7 days of anaerobic fermentation were detected. Supplementary Figure S1A shows that the amount of SCOD in the DBD group is higher than that in the control group on the 7th day of anaerobic fermentation. When the DBD discharge power was 36.80, 76.50, and 113.20 W, the SCOD concentration in sludge was 2818.60, 3209.30, and 2580.10 mg/l respectively, which was 1.40, 1.60, and 1.30 times that of the control group. The value of S_VSS_ in the control group was 26.20%, and the value of S_VSS_ in the DBD group was 30.80, 31.00, and 30.10%, respectively, when the discharge power was 36.80, 76.50, and 113.20 W (Supplementary Figure S1B). The concentration of NH+ 4-N in the control group was 254.10 mg/l, while increased to 292.80, 318.20, and 301.80 mg/l when the DBD discharge power was 36.80, 76.50, and 113.20 W (Supplementary Figure S1C). Therefore, different pretreatment discharge power will affect the solubilization and hydrolysis of organic matter in the subsequent sludge anaerobic fermentation process. With the increase of discharge power, the destruction degree of sludge structure increases, which is more conducive to the dissolution and hydrolysis of sludge. Therefore, SCOD, NH+ 4-N, and S_VSS_ gradually increase. However, when the discharge power continues to rise to 113.20 W, the microorganisms in the sludge are seriously damaged, which is not conducive to the role of microorganisms in the anaerobic fermentation process (Lu et al., 2012; Li et al., 2015).

**Figure 3:**
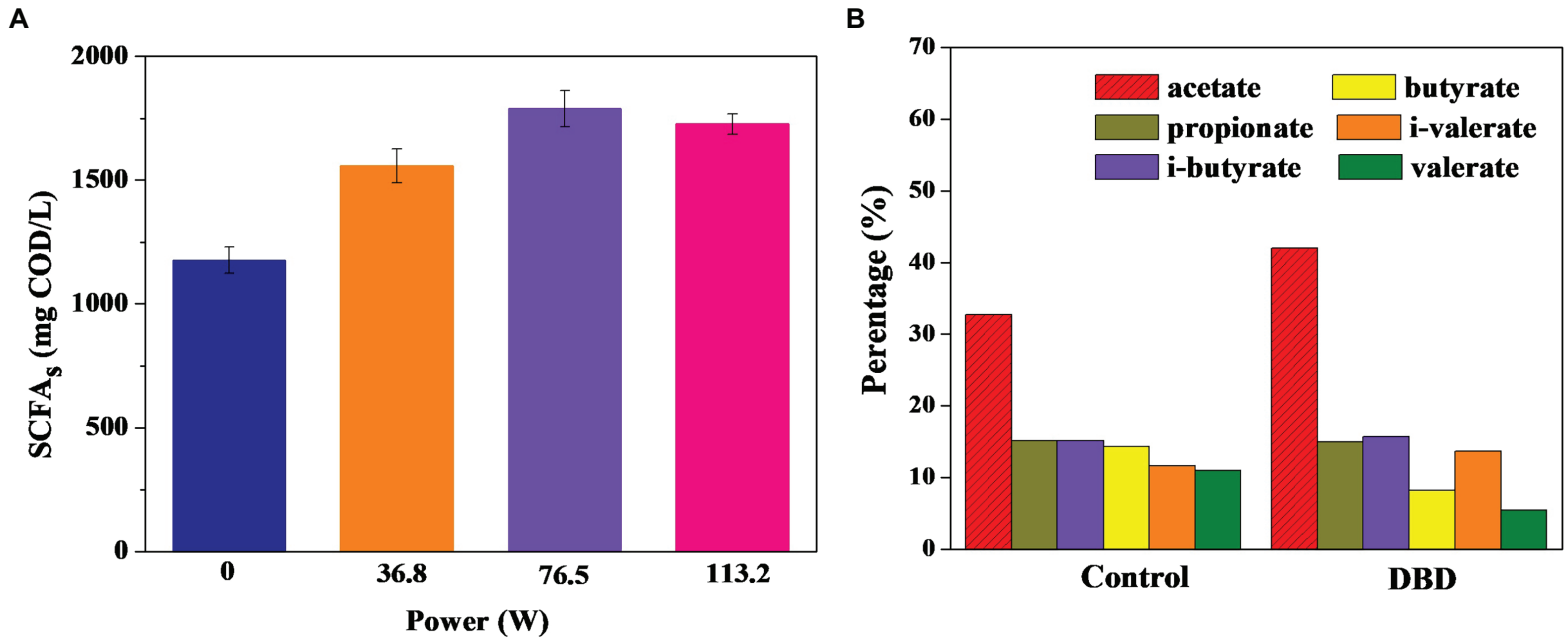
Changes in short-chain fatty acids (SCFAs) production **(A)** and acetate acid ratio **(B)** of sludge anaerobic digestion (pretreatment time is 30 min, anaerobic digestion time is 7 days).

The production of SCFAs in sludge anaerobic fermentation was further studied. Figure 3A shows that DBD pretreated sludge can promote the sludge anaerobic fermentation process to produce SCFAs. Significance analysis shows that there are significant differences between the four groups of data (*p* < 0.05). When the discharge power is 76.50 W, the concentration of SCFAs in the fermentation broth is the highest, which is 1.50 times that of the control group. Therefore, DBD pretreatment can increase the accumulation of SCFAs in anaerobic sludge fermentation. When the fermentation supernatant is recovered in the nitrogen and phosphorus removal process of sewage treatment plant, the composition of SCFAs is considered to be a key factor (Feng et al., 2009), so it is necessary to further analyze the percentages of six kinds of individual SCFAs. The composition of SCFAs in the control and DBD groups (76.50 W) was further compared and analyzed (Figure 3B). The acetic acid ratio in the DBD group was 42.00%, while it is 32.70% in the control group. Since acetic acid has been proved to be a more ideal carbon source than other kinds of acids in the denitrification process (Lee et al., 2014; Liu et al., 2016), the DBD treatment can be used as a preferred technique for the improvement of fermentation liquor quality. However, the mechanisms of SCFA accumulation in anaerobic fermentation need to be further studied.

### Effect of dielectric barrier discharge pretreatment on organic matter in sludge

**Figure 4:**
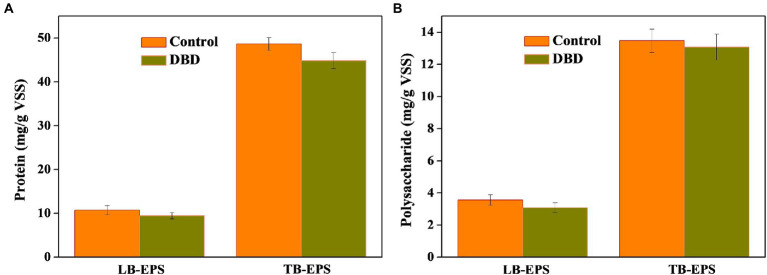
Changes in protein **(A)** and polysaccharide **(B)** concentration in loosely bound extracellular polymers (LB-EPS) and tightly bound extracellular polymers (TB-EPS) after dielectric barrier discharge (DBD) pretreatment (DBD discharge power is 76.5 W, pretreatment time is 30 min).

As we know, EPS is wrapped outside microorganisms and has a protective effect on microorganisms (Zhen et al., 2017; Liu et al., 2020). The detection of EPS is helpful to analyze the solubilization of organic substances in sludge. As can be seen in Figure 4, after DBD pretreatment, the contents of protein and polysaccharide in LB-EPS and TB-EPS decreased to varying degrees. It is speculated that DBD pretreatment can destroy EPS structure and release organic substances such as protein and polysaccharide in EPS. The cracking of EPS is conducive to the attack of DBD pretreatment on the microbial cell structure, releasing intracellular organic matter, and providing favorable conditions for the hydrolysis and acidification of the subsequent anaerobic fermentation process.

**Figure 5:**
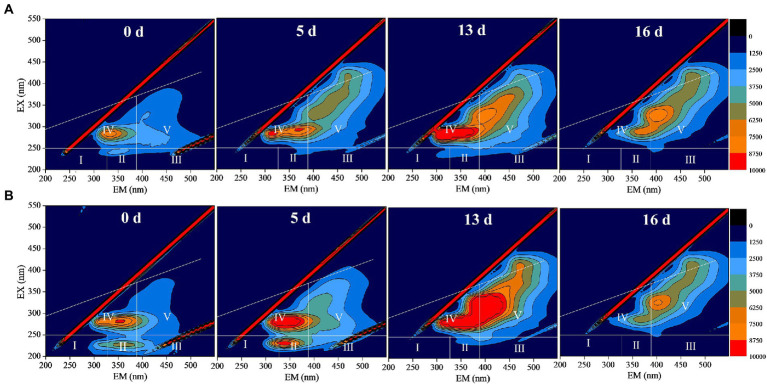
The three-dimensional fluorescence spectrum of dissolved organic matter (DOM) during sludge anaerobic digestion (**A**: no pretreatment; **B**: dielectric barrier discharge (DBD) pretreatment, DBD discharge power was 76.5 W, pretreatment time was 30 min).

Through the three-dimensional fluorescence spectrum analysis of DOM in anaerobic fermentation process, the cracking of sludge structure and the change of organic substance can be qualitatively reflected. Figures 5A,B are the three-dimensional fluorescence spectra of DOM during anaerobic fermentation for 0, 5, 13, and 16 days in the control and DBD group, respectively. Tyrosine proteins represented by region I (EX/EM = 200 ~ 250/200 ~ 330) and soluble microbial by-products represented by Region IV (EX/EM = 250 ~ 350/200 ~ 380) are generally considered to be biodegradable organics (Chen et al., 2003; Wen et al., 2015). After DBD pretreatment, the fluorescence intensity of region I increased slightly, while the fluorescence intensity of Region IV increased significantly, indicating that DBD pretreatment can improve the release of organic substances in sludge. With the progress of anaerobic fermentation of sludge, the fluorescence intensity of area IV increases first and then decreases with the increase of anaerobic fermentation time. This is because during anaerobic fermentation process, organic substances are in the dynamic change of dissolution and consumption. In the later stage of anaerobic fermentation, the quality of released organic matter is lower than the consumption, so the fluorescence intensity decreases gradually. All in all, the three-dimensional fluorescence spectrum indicated that the DBD technique can enhance the solubility of organic matters thus accelerate the biotransformation of the released organic substance.

### Analysis of microbial community structure

#### Bacterial community analysis

**Figure 6:**
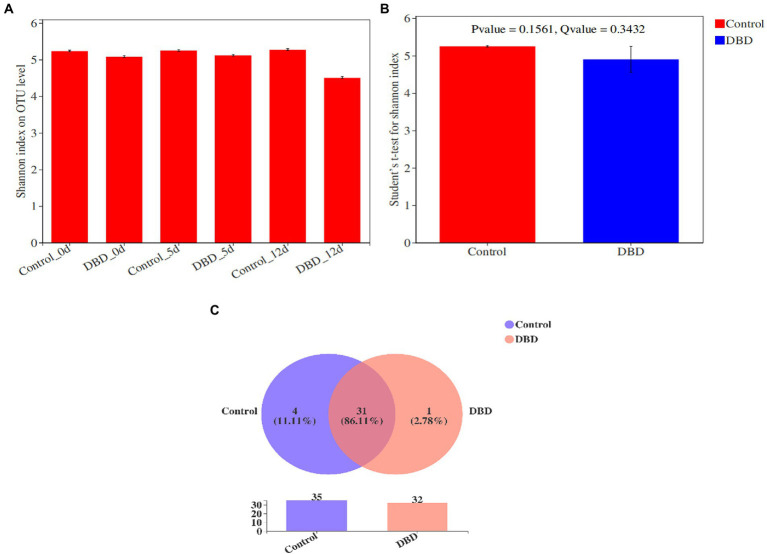
The Shannon index of microorganisms in control and dielectric barrier discharge (DBD) group **(A)**; the intergroup difference analysis of Shannon index shows that there is no significant difference between the control group and DBD group **(B)**; the Venn diagram analysis of the control and DBD group based on OTUs **(C)**.

Shannon index can be used to evaluate the microbial diversity of the community. The greater the Shannon value, the higher the diversity of the community. As shown in Figure 6A, the Shannon index of microorganisms in raw sludge was 5.23. After DBD treatment, the Shannon index decreased to 5.08. On the 5th day of anaerobic fermentation, the Shannon indexes of control group and DBD group were 5.24 and 5.12, respectively. On the 12th day of anaerobic fermentation, the Shannon indexes of blank group and DBD group were 5.27 and 4.50, respectively. Therefore, it can be concluded that DBD pretreatment will reduce the diversity of microbial communities in sludge. This is because DBD pretreatment will lead to the death of some microorganisms in sludge. However, the results of intergroup difference analysis of Shannon index show that there is no significant difference between the control group and DBD group (Figure 6B). Therefore, although DBD treatment can reduce the diversity of microbial community in sludge, this reduction is not obvious. Venn chart is used to represent the overlap of different samples and the number of unique species (such as OTU), so it can be used to characterize the similarity and overlap of species (such as OTU) composition in different samples. Figure 6C shows Venn diagram analysis of the control group and DBD group based on OTUs. The results show that the proportion of OTUs shared by the control group and DBD group in the total OTUs is as high as 86.11%. Therefore, DBD pretreatment does not significantly change the types of sludge microorganisms.

**Figure 7:**
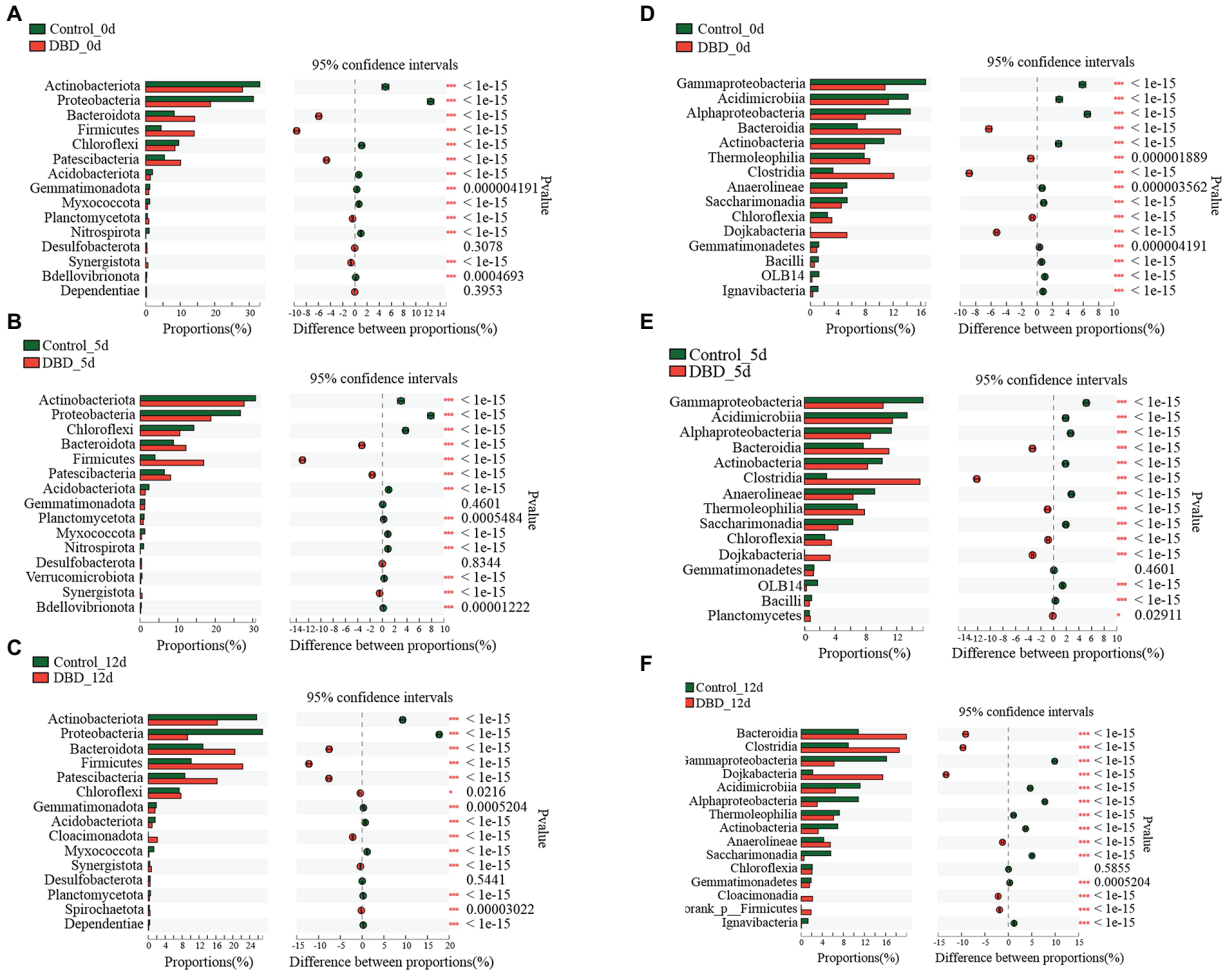
Fisher’s exact test bar plot on Phylum level: **(A)** Control and dielectric barrier discharge (DBD) test at 0 days; **(B)** Control and DBD test at 5 days; **(C)** Control and DBD test at 12 days; Fisher’ exact test bar plot on Class level: **(D)** Control and DBD test at 0 days; **(E)** Control and DBD test at 5 days; **(F)** Control and DBD test at 12 days.

Further, the distribution of bacteria at phylum and class levels in the sludge system of control and DBD group was analyzed in detail (Figure 7). Firstly, compare the abundance of species composition between different samples, that is, according to the community abundance data, the species with different microbial community abundance in different samples were selected by statistical methods, and the significance of the difference was evaluated by hypothesis test. According to Figure 7, there were significant differences in the abundance of Actinobacteriota, Proteobacteria, Bacteroidota, Firmicutes, and so on between the control group and the DBD group on days 0, 5, and 12.

**Figure 8:**
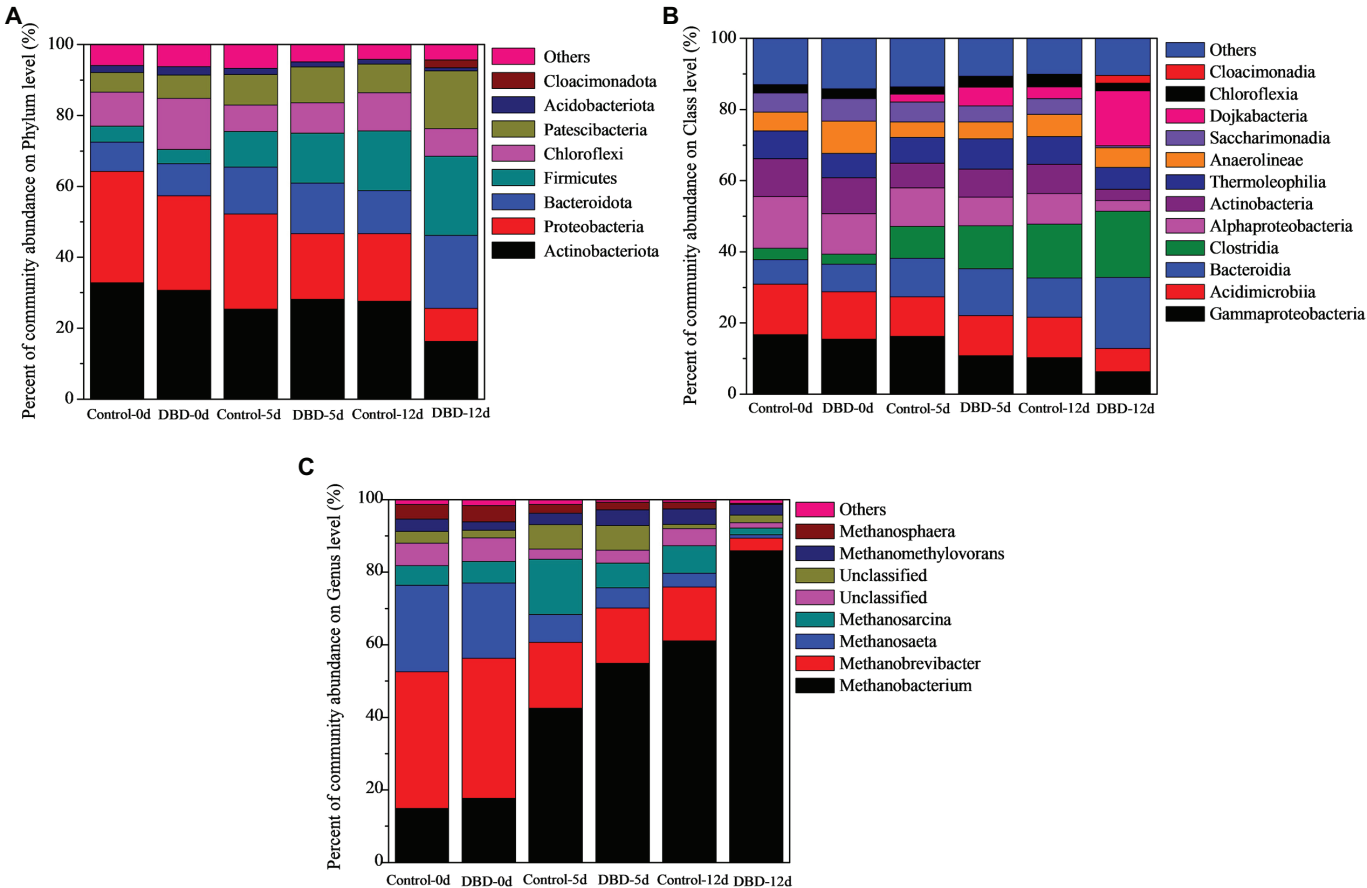
The percentage of community abundance of bacterial on phylum **(A)** and class **(B)** level during sludge anaerobic fermentation process; the percentage of community abundance of archaea on genus level during sludge anaerobic fermentation process **(C)**.

In Figure 8A, the proportion of Firmicutes increased from 4.50, 3.94, and 10.15% in the control group to 14.07, 16.94, and 22.37% in the DBD group on the 0, 5, and 12 days of anaerobic fermentation. Many studies have shown that there are a large number of Firmicutes in anaerobic fermentation reactors (Lim et al., 2014; Li et al., 2015; Zhou et al., 2020). When the concentration of released organic substrates increases, they will enrich rapidly, and can produce protease, cellulase, lipase, and other extracellular enzymes to degrade organic substances, thus affecting the formation of SCFAs (Lu et al., 2012; Zhou et al., 2020). In addition, the abundance of Bacteroidetes increased from 8.27, 8.88%, and 12.94 in the control group to 14.21, 12.21, and 20.51% in the DBD group on the 0, 5, and 12 days of anaerobic fermentation. Bacteroidetes are closely related to the metabolism of organic substances such as protein, lipids, cellulose, sugars, and amino acids in the anaerobic reactor (Hanreich et al., 2013). In addition, there are a large number of acetic acids producing bacteria in Bacteroidetes (Lu et al., 2012; Hanreich et al., 2013). In this study, the content of acetic acid in the DBD group was higher than that in the control group, which may be related to the increase of Bacteroidetes abundance. Moreover, Proteobacteria decreased from 31.22, 26.72%, and 27.04 in the control group to 18.78, 18.84, and 9.31% in the DBD group on the 0, 5, and 12 days of anaerobic fermentation. Proteobacteria is a common bacterium in anaerobic digestion reactors. Studies have shown that Proteobacteria includes many pathogens, such as Salmonella, *Helicobacter pylori*, *Vibrio cholerae,* and so on (An et al., 2009). It means that DBD pretreatment can reduce the content of harmful bacteria in sludge, so as to reduce the damage to the environment caused by sludge in subsequent treatment and disposal. Research also shows that Proteobacteria is a consumer of acids (acetic acid, propionic acid, and butyric acid), and the reduction of Proteobacteria content will be conducive to acid accumulation (Luo et al., 2018).

The community structure of bacteria was further analyzed at the class level (Figure 8B). There were significant differences in Bacteroidia, Clostridia, Alphaproteobacteria, and Gammaproteobacteria between the control group and the DBD group. On days 0, 5, and 12 of anaerobic fermentation, the content of Bacteroidia in DBD group was 1.92, 1.44, and 1.84 times higher than that in the control group, the content of Clostridia in the DBD group was 3.70, 5.20, and 2.08 times higher than that in the control group. Bacteroidia belongs to Bacteroidetes which has the ability to produce butyric acid and acetic acid. The increase of Bacteroidia abundance may be one of the reasons for the increase of acetic acid proportion in the DBD group. Clostridia belongs to Firmicutes, which can degrade carbohydrates into small molecular organics (Fu et al., 2015), and then acidogenic bacteria can degrade these small molecular organics into acetic acid, propionic acid, butyric acid, etc. In addition, AlphaProteobacteria and GammaProteobacteria belong to Proteobacteria, and their proportion in the DBD treatment group is lower than that in the control group. The decrease in their content is conducive to acid accumulation.

#### Archaeal community analysis

Anaerobic fermentation process of sludge can be divided into three stages: hydrolysis, acidification, and methanation (Liang et al., 2021; Wu et al., 2021). SCFA is the product of acidification and methane is the product of methanogenesis (Garcia et al., 2000; Lee et al., 2014). Therefore, the production of methane will affect the accumulation of SCFAs. Archaea is closely related to methane production (Jiang et al., 2021a); therefore, the analysis of Archaea community structure will help to clarify the mechanism of sludge fermentation and SCFAs production. Previous studies have shown that methanogenic microorganisms can be divided into two categories according to the type of substrate consumed: one is to produce methane by using acetic acid and the other is to produce methane by using H_2_/CO_2_ (Garcia et al., 2000; Demirel and Scherer, 2008). *Methanosaeta* sp. and *Methanosarcina sp.* are obligate acetate-utilizing methanogens (Demirel and Scherer, 2008); therefore, as can be seen from Figure 8C, the sum of abundance percentage of *Methanosaeta sp.* and *Methanosarcina sp.* in raw sludge was 29.20%. After DBD pretreatment of sludge, this value decreased to 26.70%. This means that DBD pretreatment has a certain degree of damage to the activity of *Methanosaeta sp.* and *Methanosarcina sp.* With the progress of anaerobic fermentation, on the 5th day of anaerobic fermentation, the sum of the community abundance percentages of *Methanosaeta sp.* and *Methanosarcina sp.* in the control group decreased to 22.90%, while in the DBD group, it was as low as 12.40%. We can speculate that the decrease of *Methanosaeta* sp. and *Methanosarcina sp*. abundance in the DBD group may be another reason for the increase in acetic acid accumulation.

## Conclusion

This study demonstrates that DBD technique is an effective pretreatment method to enhance sludge anaerobic fermentation performance. The highest accumulation of SCFAs in the fermentation broth was observed on the 7th day of anaerobic fermentation when the DBD power was 76.50 W, which was 1.50 times of the control. The percentage of acetic acid in the DBD group was 9.30% higher than that in the control. Mechanism research shows that the DBD technique could enhance the release of biodegradable organics, so as to facilitate the biotransformation of organic matter. The increase in the abundance of Firmicutes and Bacteroidetes and the decrease in the abundance of Proteobacteria in DBD group were beneficial to SCFA accumulation. Besides, the decrease of *Methanosaeta sp*. and *Methanosarcina sp*. abundance in the DBD group facilitate acetic acid accumulation.

## Data availability statement

The original contributions presented in the study are included in the article/Supplementary material, further inquiries can be directed to the corresponding author.

## Author contributions

JW: funding acquisition, methodology, and writing—original draft. XL: supervision, project administration, funding acquisition, and writing—reviewing and editing. JH: experimental work. GC: writing—reviewing and editing. JX: investigation, data analysis, and writing—reviewing and editing. ML: software and formal analysis. YS: formal analysis. AZ: conceptualization and methodology. All authors contributed to the article and approved the submitted version.

## Funding

This work was supported by the National Key Research and Development Program of China (nos. 2019YFD0900301, 2020YFD0900405, and 2020YFD0900401), the Natural Science Foundation of Shanghai (no. 21ZR1479900), Modern Agricultural Industrial Technology system in China (no. CARS-46), and Shanghai Agriculture Applied Technology Development Program, China (no. T20210301).

## Conflict of interest

The authors declare that the research was conducted in the absence of any commercial or financial relationships that could be construed as a potential conflict of interest.

## Publisher’s note

All claims expressed in this article are solely those of the authors and do not necessarily represent those of their affiliated organizations, or those of the publisher, the editors and the reviewers. Any product that may be evaluated in this article, or claim that may be made by its manufacturer, is not guaranteed or endorsed by the publisher.

## Supplementary material

The Supplementary material for this article can be found online at: https://www.frontiersin.org/articles/10.3389/fmicb.2022.963260/full#supplementary-material

## Footnotes

^1^ http://www.ncbi.nlm.nih.gov/sra/PRJNA827336
