# Supplementary material for: Mechanism of dielectric barrier discharge plasma technology to improve the quantity of short-chain fatty acids in anaerobic fermentation of waste active sludge
Source: Front Microbiol. 2022 Jul 22;13:963260. doi: 10.3389/fmicb.2022.963260 (PMC9355127; doi:10.3389/fmicb.2022.963260)
Supplement: Supplementary file 1 [file Presentation_1.pdf]

Mechanism of dielectric barrier discharge plasma technology to improve the quantity of short chain fatty acids in anaerobic fermentation of waste active sludge

Jie Wang[1,2], Xingguo Liu[1,2], Jinling He[3], Guofeng Cheng[1,2], Junli Xu[6], Ming Lu[5], Yuyi Shangguan[4], Ai Zhang[3]*

[1]Fishery Machinery and Instrument Research Institute of Chinese Academy of Fishery Sciences, 63 Chifeng Road, Shanghai, 200092, China;

[2] Key Laboratory of aquaculture facilities engineering, Ministry of agriculture and rural affairs, 63 Chifeng Road, Shanghai, 200092, China;

[3] College of Environmental Science and Engineering, Donghua University, 2999 North Renmin Road, Shanghai 201620, China;

[4] School of Ecological and Environmental Sciences, East China Normal University, 500 Dongchuan Road, Shanghai, 200241, China;

[5] School of Environment and Architecture, University of Shanghai for Science and Technology, 516 Jungong Road, Shanghai, 200093, China;

[6] School of Ecology and Environment, Yellow River Conservancy Technical Institute, No. 1 Dongjing Road, Kaifeng, 475004, Henan Province, China.

**Corresponding author: Tel: +86 21 67792538; Fax: +86 21 67792522; E-mail: aizhang@dhu.edu.cn*

Text S1

Dielectric barrier plasma equipment

The plate type dielectric barrier discharge reactor is mainly composed of a low-temperature plasma reactor (positive and negative electrodes) and a low-temperature plasma reaction dish (quartz reaction dish). The upper electrode is connected to the high-voltage power supply and the lower electrode is grounded.

Text S2

EPS extraction

The sludge sample was centrifuged at 3000 g for 10 min and abandon the supernatant. Dissolve the remaining substance with 15 mL of 0.05% NaCl solution, sonicate for 2 min, shake on a shaking table of 150 rmp for 10 min, continue sonication for 2 min, and centrifuge at 5000 g for 10 min to separate the supernatant as LB-EPS. The remaining material was dissolved in 0.05% NaCl, sonicated for 3 min, heated at 60 ^{o}C for 30 min, then centrifuged at 9000 g for 15 min, collect the supernatant as TB-EPS. All collected supernatants passed 0.45 μm cellulose acetate membrane filtration for analysis.

Table S1 Basic characters of the waste activated sludge

TS（g/L）	pH	TSS（g/L）	VSS（g/L）	SCOD（mg/L）
19.8 ± 0.9	7.4 ± 0.3	18.6 ± 0.5	10.83 ± 0.6	130 ± 37

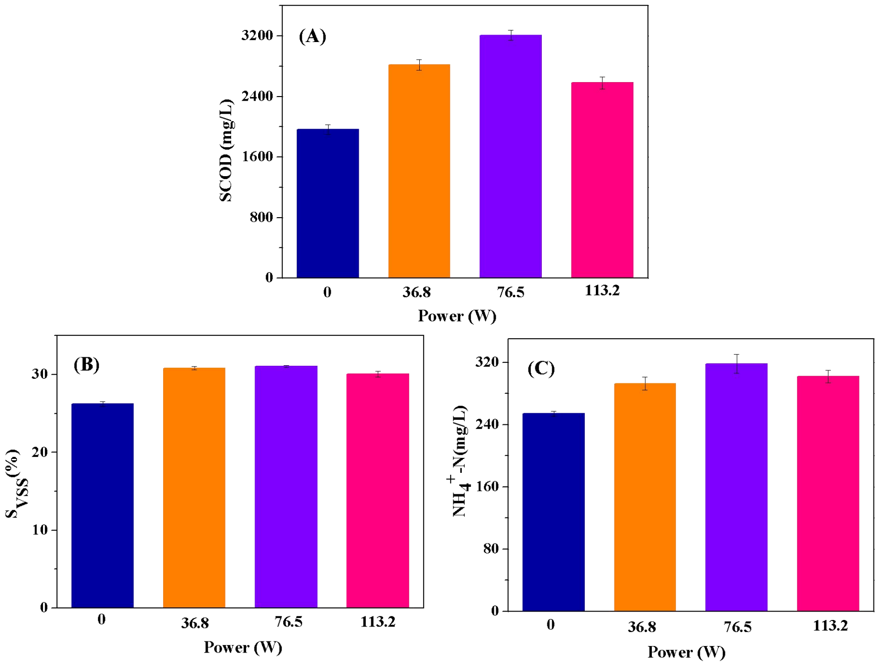

Figure S1 Changes of soluble chemical oxygen demand (SCOD) (A), the VSS solubilization rate (S_{VSS}) (B), and ammonia nitrogen (NH_4^+-N) concentration in sludge supernatant (C) during anaerobic digestion of sludge (pretreatment time is 30 min, anaerobic digestion time is 7 d)
